# *PLAUR *polymorphisms and lung function in UK smokers

**DOI:** 10.1186/1471-2350-10-112

**Published:** 2009-10-31

**Authors:** Ceri E Stewart, Ian P Hall, Stuart G Parker, Miriam F Moffat, Andrew J Wardlaw, Martin J Connolly, Charlotte Ruse, Ian Sayers

**Affiliations:** 1 Division of Therapeutics & Molecular Medicine, Nottingham Respiratory Biomedical Research Unit, University Hospital of Nottingham, Nottingham, UK; 2 Sheffield Institute for Studies on Ageing, University of Sheffield, Barnsley Hospital NHSFT, Barnsley, UK; 3 National Heart and Lung Institute, Imperial College, London, UK; 4 Institute for Lung Health, Immunity and Inflammation, University of Leicester, Leicester, UK; 5 Freemasons' Department of Geriatric Medicine, University of Auckland, Auckland, New Zealand; 6 Sheffield Institute for Studies on Ageing, University of Sheffield, Community Sciences Centre, Northern Hospital, Sheffield, UK

## Abstract

**Background:**

We have previously identified Urokinase Plasminogen Activator Receptor (*PLAUR*) as an asthma susceptibility gene. In the current study we tested the hypothesis that *PLAUR *single nucleotide polymorphisms (SNPs) determine baseline lung function and contribute to the development of Chronic Obstructive Pulmonary Disease (COPD) in smokers.

**Methods:**

25 *PLAUR *SNPs were genotyped in COPD subjects and individuals with smoking history (n = 992). Linear regression was used to determine the effects of polymorphism on baseline lung function (FEV_1_, FEV_1_/FVC) in all smokers. Genotype frequencies were compared in spirometry defined smoking controls (n = 176) versus COPD cases (n = 599) and COPD severity (GOLD stratification) using logistic regression.

**Results:**

Five SNPs showed a significant association (p < 0.01) with baseline lung function; rs2302524(Lys220Arg) and rs2283628(intron 3) were associated with lower and higher FEV_1 _respectively. rs740587(-22346), rs11668247(-20040) and rs344779(-3666) in the 5'region were associated with increased FEV_1_/FVC ratio. rs740587 was also protective for COPD susceptibility and rs11668247 was protective for COPD severity although no allele dose relationship was apparent. Interestingly, several of these associations were driven by male smokers not females.

**Conclusion:**

This study provides tentative evidence that the asthma associated gene *PLAUR *also influences baseline lung function in smokers. However the case-control analyses do not support the conclusion that *PLAUR *is a major COPD susceptibility gene in smokers. PLAUR is a key serine protease receptor involved in the generation of plasmin and has been implicated in airway remodelling.

## Background

Chronic Obstructive Pulmonary Disease (COPD) and asthma are complex respiratory diseases involving both genetic and environmental factors (*e.g*. smoking exposure in COPD, allergen exposure in asthma) [1,2]. Using 587 asthma families we have recently fine mapped a 14.4 Mb region on Chromosome 19q13 and identified the urokinase plasminogen activator receptor (*PLAUR*, plasminogen activator receptor, urokinase type, alternative symbols; *UPAR *and *CD87*) gene as an asthma susceptibility gene [3]. Importantly, we also demonstrated that polymorphisms spanning *PLAUR *predict decline in forced expiratory volume in 1 second (FEV_1_) in asthma subjects and determine plasma PLAUR levels [3]. PLAUR plays a key role in the formation of the serine protease plasmin by interacting with urokinase plasminogen activator (PLAU) [4] and has been implicated in many processes including; cell differentiation, proliferation and migration [5]. Plasminogen activator inhibitors (PAI-1 and PAI-2) regulate PLAUR activity [6].

While asthma and COPD are distinct clinical entities, a common feature of both diseases is airway remodelling, *i.e*. deposition of extracellular matrix (ECM) in the submucosa, thickening of the reticularis and smooth muscle hyperplasia [7,8]. From the known biology of PLAUR, this protease receptor is potentially involved in these processes due to its role in matrix metalloproteinase (MMP) and transforming growth factor (TGF)β1 activation and tissue fibrosis [5].

Whole genome linkage analyses using the Boston early onset COPD family cohort has identified linkage to chromosome 19q13 for FEV_1_, FVC and FEV_1_/FVC, LOD 1.40 (59 cM), 1.20 (101 cM), 1.47 (61 cM) respectively [9]. Typing additional markers on 19q13 provided further support for this region *i.e*. FEV_1 _and FEV_1_/FVC LOD 1.73 (62 cM), 1.70 (62 cM) which was strengthened by selection of former/current smokers, LOD 3.30 (71 cM) and 1.96 (63 cM) respectively [10]. The *PLAUR *gene is located at 67 cM and based on genetic and biological evidence may be a COPD susceptibility gene. Smoking is associated with an increased decline in FEV_1 _and is a major risk factor for the development of COPD [11] therefore we investigated the role of *PLAUR *SNPs in smokers.

The aim of the current study was to test the hypotheses that a) polymorphisms spanning *PLAUR *contribute to COPD susceptibility and severity in smokers and b) that these polymorphisms influence baseline lung function (FEV_1 _and FEV_1_/FVC) in smokers. We have genotyped 27 SNPs spanning *PLAUR *in a cohort recruited for COPD or smoking history (n = 992 subjects) and completed a series of association analyses. We provide tentative evidence that polymorphisms spanning *PLAUR *influence baseline lung function but do not support the conclusion that *PLAUR *is a major COPD susceptibility gene in smokers.

## Methods

### Subjects and Clinical Assessment

The subjects were recruited from five UK centres for smoking history and/or COPD diagnosis (Table 1). 537 subjects were collected in Nottingham, Caucasian > 40 years and smoking >10 pack years or other centres (n = 455) recruited for spirometry defined COPD confirmed by a physician, Caucasian > 45 years old and smoking >10 pack years. The combined population (n = 992) recruited for smoking history or COPD diagnosis were stratified into healthy smokers (n = 176, post bronchodilator (BD, salbutamol) FEV_1 _> 80% and postBD FEV_1_/FVC > 0.7) and COPD subjects (n = 599, post BD FEV_1 _< 80% and postBD FEV_1_/FVC < 0.7). Subjects without data or not meeting these criteria were excluded from the case control analyses. COPD severity was investigated using post bronchodilator spirometry, *i.e*. GOLD classification [12] (n = 643, Additional file 1). Ethical approval was obtained from local ethics committees (Nottingham, Sheffield, Manchester, Leicester, Oxford). Informed Consent from all subjects was obtained.

**Table 1:** Baseline characteristics of study cohorts

	Smokers (n = 992)	Controls (n = 176)	COPD (n = 599)	Male Smokers (n = 553)	Female Smokers (n = 431)
Age in years (mean ± SD)	63.33 ± 10.28 (992)	54.38 ± 9.52	65.96 ± 9.01	64.98 ± 10.01	61.23 ± 10.32
Female (%)	43.8 (984)	56.3	40.4	0	100
% predicted FEV_1_ (mean ± SD)	56.01 ± 28.17 (975)	96.03 ± 12.15	40.31 ± 15.63	52.82 ± 26.87	60.24 ± 29.43
FEV_1_/FVC Ratio (mean ± SD)	55.3 ± 17.4 (971)	77.3 ± 5.9	46.3 ± 12.6	53.7 ± 17.3	57.5 ± 17.5
Post BD % predicted FEV_1_ (mean ± SD)	59.08 ± 27.14 (885)	99.48 ± 11.72	44.65 ± 15.52	55.34 ± 25.46	64.10 ± 28.55
Post BD FEV_1_/FVC Ratio (mean ± SD)	55.6 ± 17.7 (873)	79.1 ± 5.5	46.2 ± 12.0	53.2 ± 17.6	58.6 ± 17.6
Pack Years (mean ± SD)	43.52 ± 26.05 (969)	32.74 ± 20.04	47.61 ± 27.01	48.72 ± 28.53	36.65 ± 20.66

FEV_1 _Forced expiratory volume in one second, FVC forced vital capacity. Number in brackets represents data available for that variable. Continual variables between Controls and COPD groups were compared by independent T-Test, categorical variables by Pearson chi square (all variables p < 0.0001). Similarly, all variables between males and females were statistically different.

### SNP selection and Genotyping

The Human *PLAUR *gene has been sequenced in 46 Caucasian individuals (SeattleSNPs, http://pga.mbt.washington.edu/). SNPs were chosen for inferred function or their ability to tag Linkage Disequlibrium (LD) blocks ([3], Table 2). CEPH genotyping data from the HapMap project (B34) in conjunction with Haploview software (v3.3) was used to identify tagging SNPs within the gene (R^2 ^0.8, Minor Allele Frequency (MAF) 0.1) or 5'distal region (R^2 ^0.75, MAF 0.2) [13]. SNPs were genotyped by Kbiosciences (Hitchin, UK) using genomic DNA. Hardy-Weinberg Equilibrium was assessed using Haploview software [13].

**Table 2:** Gene location and minor allele frequencies of *PLAUR *SNPs genotyped

**No**.	SNP	Location in PLAUR	Alleles	Selected for:	Smokers	Controls	COPD
1	rs4803648	3'UTR	A/T	LDtSNP	0.20	0.19	0.19
2	rs4802189	3'UTR	A/C	LDtSNP	0.16	0.16	0.15
3	rs4251953	3'UTR/intron	G/A	LDtSNP	0.04	0.05	0.04
4	rs4251938	3'UTR/intron	A/G	LDtSNP	0.12	0.12	0.11
5	rs4251923	3'UTR/intron	G/A	3'UTR	0.04	0.02	0.04
6	rs4760	Exon 7	T/C	Pro317/272Leu, LDtSNP	0.16	0.17	0.15
7	rs2302524	Exon 6	T/C	Lys220/175Arg LDtSNP	0.16	0.16	0.15
8	rs4251864	Intron 3	T/C	LDtSNP	0.09	0.09	0.08
9	rs2239372	Intron 3	G/A	LDtSNP	0.50	0.48	0.51
10	rs2283628	Intron 3	T/C	LDtSNP	0.18	0.18	0.18
11	rs4251846	Intron 3	C/T	LDtSNP	0.12	0.11	0.13
12	rs2239374	Intron 3	C/T	LDtSNP	0.18	0.19	0.17
13	rs2286960	Intron 1	C/T	LDtSNP	0.24	0.28	0.23
14	rs4251805	5'UTR-119	G/A	Promoter	0.04	0.03	0.04
15	rs344781	5'UTR-466	T/C	Promoter	0.23	0.29	0.29
16	rs2356338	5'UTR-649	G/T	Promoter	0.28	0.29	0.29
17	rs344780	5'UTR-3545	C/T	Promoter	0.23	0.20	0.22
18	rs344779	5'UTR-3666	G/T	LDtSNP	0.39	0.40	0.37
19	rs8113334	5'UTR-6873	T/C	LDtSNP	0.19	0.19	0.19
20	rs4493171	5'UTR-10534	C/T	LDtSNP	0.22	0.19	0.23
21	rs7259340	5'UTR-12331	C/A	LDtSNP	0.36	0.37	0.34
22	rs11668247	5'UTR-20040	C/T	LDtSNP	0.39	0.43	0.37
23	rs346043	5'UTR-20459	T/C	LDtSNP	0.26	0.26	0.27
24	rs740587	5'UTR-22346	T/C	LDtSNP	0.45	0.49	0.42
25	rs346054	5'UTR-30147	C/G	LDtSNP	0.45	0.47	0.46

				Number	**992**	**176**	**599**

LDtSNP = LD tagging SNP

### Statistical Analyses

Using SPSS (version 15, SPSS Inc., Chicago, IL) logistic regression analyses were completed for dichotomous traits using additive (*e.g*. AA vs. AC vs. CC), recessive (AA/AC vs. CC) or dominant (AA vs. AC/CC) models. The COPD susceptibility analyses included, age, sex and pack years as covariates (Table 1) and the GOLD 1 versus 4 analyses included age as a covariate. Unadjusted contingency table analyses were completed using genotype or allele models (GraphPad Prism version 5, San Diego, CA). Linear regression determined the contribution of each SNP to baseline FEV_1 _(litres) or FEV_1_/FVC ratio using additive, recessive or dominant models including; age, sex, height and smoking pack years as covariates. Haploview software was used to identify *PLAUR *haplotypes. A p < 0.01 was considered significant for all analyses.

### 5'region analyses

Basal promoter activity of *PLAUR *has been mapped to 220 bp upstream of the start codon [14]. Using DNA from 31 Caucasian subjects a 4 kb promoter region was amplified and sequenced (Applied Biosystems, UK). SNPs were analysed for transcription factor (TF) binding site changes using online databases as described [15].

## Results

### Clinical Characteristics and Allele Frequencies

Baseline features of the COPD cases (n = 599) and controls (n = 176) are shown in Table 1. As anticipated baseline FEV_1 _and FEV_1_/FVC of the smoking controls and COPD cases are significantly different (p < 0.0001). Comparison of other baseline features between controls and cases identified significant differences for age, sex and pack years, therefore in subsequent analyses we adjusted for these variables. Also shown are the baseline features of male and female smokers which show that females have significantly less smoking exposure and increased lung function compared to male subjects (p < 0.001). SNPs spanning *PLAUR *were genotyped (Table 2). Two of the 27 SNPs, rs1994417 and rs4251831 showed deviation from Hardy-Weinberg equilibrium (p = 0.028 and p = 0.004 respectively) in the entire population (n = 992). These SNPs were removed from subsequent analyses.

### Haplotype structure

**Figure 1:**
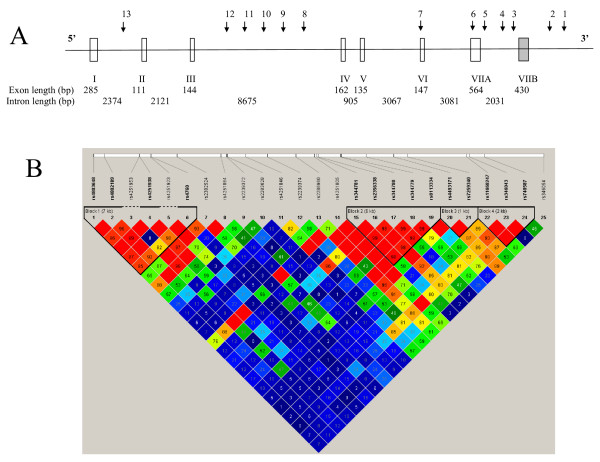
**Schematic representation of the *PLAUR *gene and haplotype block structure of *PLAUR *SNPs in smokers**. (A) Schematic representation of the *PLAUR *gene on chromosome 19 illustrating the position of SNPs 1-13 (see Table 2). SNPs 14-25 located in the promoter region/5'region are omitted for clarity. The *PLAUR *gene is displayed in the reverse orientation to that observed on chromosome 19 (~40 kb). Exons are depicted as open boxes except for alternatively spliced exon 7 (VIIB, grey, see [34]). (B) Haplotype block structure of all 25 SNPs on Chromosome 19 in smokers (n = 992). The colour of shading represents R^2 ^(a measure of LD) and numerical values are given (generated using Haploview software [13]). Haplotype blocks were defined using confidence intervals for strong LD D' 0.7-0.98.

The haplotype structure of the *PLAUR *region generated using all data is shown in Figure 1. These data revealed that there is some redundancy in the genotyping (as expected) and that there is a block of Linkage Disequilibrium (LD) in the 3'region, a region of low LD spanning the gene and several blocks of LD in the 5'region (Figure 1).

### *PLAUR *SNPs and COPD susceptibility in smokers

Analyses of the smoking control cohort (n = 176) versus the COPD cohort (n = 599) identified only one SNP meeting statistical significance (p < 0.01), *i.e*. rs740587(-22346) showed a protective effect for COPD susceptibility (Table 3). Several other SNPs showed borderline significance including SNPs in intron 1 (rs2286960, protective(P)) and in the 5'region *i.e*. rs344779(-3666, P), rs11668247(-20040, P) and rs346054(-30147, P). These data provide limited evidence that *PLAUR *SNPs are associated with COPD susceptibility *per se*.

**Table 3:** Risk of COPD in smokers and *PLAUR *SNPs

	Controls (n = 176)			COPD (n = 599)			Additive			Recessive			Dominant		
SNP	0	1	2	0	1	2	p-value	Odds ratio	95%CI	p-value	Odds ratio	95%CI	p-value	Odds ratio	95%CI
rs4803648	112	48	9	381	180	21	0.963	1.01	0.70-1.45	0.313	0.60	0.23-1.61	0.637	1.11	0.72-1.70
rs4802189	117	47	4	420	151	12	0.964	0.99	0.66-1.48	0.735	0.79	0.20-3.13	0.955	1.01	0.65-1.59
rs4251953	158	15	1	540	43	2	0.800	0.92	0.49-1.74	0.825	1.38	0.08-24.45	0.742	0.89	0.45-1.77
rs4251938	133	38	1	466	116	8	0.969	0.99	0.63-1.56	0.656	1.70	0.17-17.27	0.883	0.96	0.59-1.57
rs4251923	166	8	0	544	44	0	0.598	1.28	0.52-3.16	ND	ND	ND	0.598	1.28	0.52-3.16
rs4760	122	38	10	429	146	13	0.271	0.81	0.56-1.18	0.106	0.42	0.15-1.20	0.523	0.86	0.55-1.36
rs2302524	121	54	1	422	149	16	0.691	0.92	0.63-1.37	0.081	7.23	0.78-66.86	0.293	0.79	0.51-1.22
rs4251864	143	30	1	507	81	5	0.976	1.01	0.61-1.66	0.480	2.38	0.22-26.30	0.876	0.96	0.56-1.64
rs2239372	48	84	41	150	284	156	0.679	1.06	0.80-1.40	0.857	1.04	0.66-1.66	0.621	1.12	0.71-1.76
rs2283628	122	43	10	396	172	23	0.822	0.96	0.67-1.38	0.118	0.47	0.18-1.21	0.706	1.09	0.71-1.67
rs4251846	141	31	3	452	134	8	0.518	1.16	0.74-1.82	0.380	0.50	0.11-2.34	0.340	1.27	0.78-2.08
rs2239374	115	57	4	401	173	15	0.379	0.85	0.59-1.23	0.785	0.84	0.25-2.88	0.369	0.83	0.54-1.26
rs2286960	91	71	13	347	215	30	0.022	0.68	0.49-0.95	0.049	0.45	0.20-0.99	0.061	0.68	0.45-1.02
rs4251805	163	11	0	538	46	1	0.323	1.49	0.68-3.30	ND	ND	ND	0.340	1.48	0.66-3.30
rs344781	110	50	13	358	191	33	0.719	1.06	0.76-1.49	0.729	0.86	0.38-1.97	0.534	1.14	0.75-1.74
rs2356338	90	69	16	282	271	38	0.397	1.15	0.83-1.60	0.685	0.85	0.39-1.86	0.215	1.29	0.86-1.92
rs344780	107	42	11	341	180	31	0.611	1.10	0.77-1.56	0.729	0.86	0.36-2.06	0.422	1.197	0.77-1.86
rs344779	65	78	33	229	287	74	0.097	0.78	0.58-1.05	0.029	0.54	0.31-0.94	0.437	0.85	0.57-1.28
rs8113334	120	47	9	397	172	24	0.969	1.01	0.71-1.44	0.478	0.71	0.27-1.85	0.729	1.08	0.70-1.66
rs4493171	114	53	7	348	215	28	0.204	1.26	0.88-1.79	0.768	1.16	0.44-3.09	0.165	1.34	0.89-2.04
rs7259340	70	77	26	251	263	69	0.147	0.80	0.60-1.08	0.155	0.65	0.36-1.18	0.299	0.81	0.54-1.21
rs11668247	67	68	41	241	267	87	0.034	0.74	0.56-0.98	0.022	0.55	0.34-0.92	0.172	0.75	0.50-1.13
rs346043	94	66	12	305	251	36	0.279	1.20	0.86-1.67	0.836	1.09	0.48-2.48	0.221	1.28	0.86-1.92
**rs740587**	**54**	**72**	**50**	**206**	**272**	**110**	**0.010**	**0.70**	**0.53-0.92**	**0.016**	**0.56**	**0.35-0.90**	**0.056**	**0.66**	**0.43-1.01**
rs346054	59	69	48	171	296	124	0.293	0.86	0.66-1.14	0.011	0.55	0.35-0.87	0.509	1.15	0.76-1.77

0, 1, 2 represent number of genotypes for major, heterozygote and minor genotypes respectively. For minor allele frequencies in controls versus COPD cases see Table 2. Logistic regression was used to compare genotype frequencies using the additive, recessive, and dominant models with covariates age, sex and pack years included in the model. ND = Not determined (minor allele frequency too low).

### *PLAUR *SNPs do not show association with spirometry defined COPD severity

Subjects were stratified according to GOLD criteria (Additional file 1) and unadjusted analyses comparing GOLD groups 1, 2, 3 and 4 and extreme severity (GOLD 1 versus 4, adjusted for age) were completed. GOLD Stage 1-4 analyses did not identify any SNPs showing significant association. Adjusted analyses (age) of GOLD Stage 1 and Stage 4 using logistic regression identified rs11668247(5'UTR-20040) as a protective allele (dominant model OR 0.32 CI 0.14-0.77, p = 0.011) (Additional file 1).

### *PLAUR *SNPs influence baseline lung function in smokers

**Figure 2:**
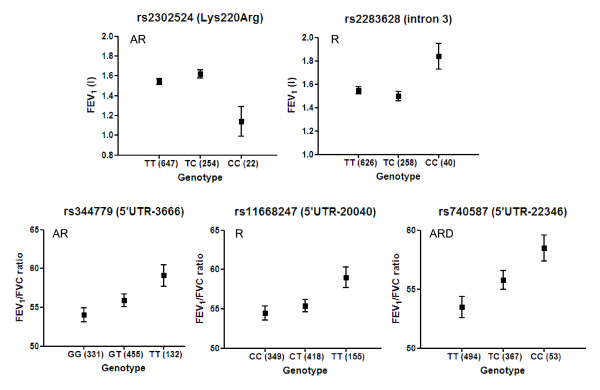
***PLAUR *SNPs are associated with baseline FEV_1 _and FEV_1_/FVC**. Regression analysis was used to investigate the association between *PLAUR *SNPs and baseline FEV_1 _and FEV_1_/FVC ratio using the additive, recessive or dominant models. Data represent mean ± standard error. Covariates included in the model included age, gender, height and pack years. A = Additive, R = Recessive, D = Dominant models showing p < 0.01 are presented.

In addition to dichotomous trait analyses based on post bronchodilator spirometry we also investigated the role of *PLAUR *SNPs in determining baseline lung function, FEV_1 _and FEV_1_/FVC ratio in the entire population (n = 992) (Figure 2 and Additional file 2). The FEV_1 _analyses identified two SNP associations; rs2302524(Lys220Arg) was associated with lower FEV_1 _and rs2283628 (intron 3) was associated with higher FEV_1 _(Figure 2). Both associations were driven by carriers of two variant alleles (recessive model p = 0.006 and p = 0.008 respectively).

In the baseline FEV_1_/FVC analyses three SNPs showed association, *i.e*. rs740587(5'UTR-22346), rs11668247(5'UTR-20040) and rs344779(5'UTR-3666) in the 5'region were significantly associated with improved FEV_1_/FVC ratio (Figure 2, Additional file 2). Borderline significance was also observed for rs344781(5'UTR-466) and rs2283628(intron 3) for higher baseline FEV_1_/FVC values and rs346043(5'UTR-204590) and rs2356338(5'UTR-649) for reduced baseline FEV_1_/FVC values. These data highlight the potential importance of genetic determinants in the 5'region particularly for the FEV_1_/FVC phenotype. In order to determine if males or females are driving these associations with lung function we completed gender specific analyses for the key associated SNPs (Additional file 2). The baseline features of the male and female only cohorts are shown in Table 1. These data suggested that both the rs740587(5'UTR-22346) and rs344779(5'UTR-3666) associations with FEV_1_/FVC are driven by males as the allele dose effect observed in all subjects is present in male subjects and is lost in females *e.g*. rs344779 FEV_1_/FVC data for males; GG 51.8 ± 1.2 (185), GT 54.5 ± 1.0 (260), TT 59.1 ± 2.0 (66) p = 0.007 versus females; GG 56.8 ± 1.4 (146), GT 57.7 ± 1.2 (195), TT 59.5 ± 1.8 (66), p = 0.531.

### *PLAUR *promoter analyses identifies novel SNPs with predicted function

As several associations were located in the 5'region we sequenced 4000 bps of the key promoter region (LD block 2, Figure 1) in 37 Caucasian subjects to identify novel variation. A further five novel and two reported polymorphism were identified (Additional file 3). The 12 polymorphisms were in high LD (data not shown) which suggested identification of causative polymorphisms will be difficult. Nonetheless, we conducted a bioinformatics analysis and three of these polymorphisms were found to generate binding sites for the E26 transforming sequence (ets) family transcription factor binding sites. Multiple potential changes in transcription factor (TF) binding sites were observed for SNPs in the 5' distal region *e.g*. alterations in hypoxia inducible factor 1 binding sites (Additional file 3).

## Discussion

This study provides preliminary evidence that SNPs within *PLAUR *influence baseline lung function in smokers, however does not support the conclusion that *PLAUR *SNPs contribute significantly to the multiple genetic factors that predispose smokers to develop COPD.

We have previously identified *PLAUR *as an asthma and lung function associated gene and in particular identified the 3'region (rs4803648, rs4802189), intron 3 (rs2239372) and 5'region (rs2356338, rs4493171, rs346043) as determinants [3]. *PLAUR *SNPs predicted decline in FEV_1 _in asthma subjects and were associated with PLAUR levels in plasma [3] (see Table 4). These data and the emerging role of PLAUR in tissue remodelling suggested *PLAUR *may also influence COPD susceptibility in smokers. To test this hypothesis we genotyped 27 SNPs spanning *PLAUR *in a cohort of 992 smokers recruited for smoking history and/or COPD diagnosis, 25 SNPs passed quality control. Data from all subjects identified multiple 5'region LD blocks, a region of low LD spanning the gene and a 3'region LD block in keeping with our previous data [3].

**Table 4:** Concordance between asthma analyses and the current study

SNP	Allele	*Asthma Susceptibility	Decline in FEV_1 _in asthma	*Serum PLAUR	COPD Susceptibility	COPD Severity (GOLD 1/4)	Baseline FEV_1_	Baseline FEV_1_/FVC
rs4803648	T	Risk	Accelerated	-	-	-	-	-
rs4802189	C	Risk	Accelerated	-	-	-	-	-
rs4251953	A	-	Accelerated	-	-	-	-	-
rs4251938	G	Risk	-	-	-	-	-	-
rs4251923	A	-	-	-	-	-	-	-
rs4760	C	-	Slowed	-	-	-	-	-
rs2302524	C	Risk	Accelerated	-	-	-	Decreased	-
rs4251864	C	-	-	-	-	-	-	-
rs2239372	A	Risk	-	Increased	-	-	-	-
rs2283628	C	-	-	-	-	-	Increased	-
rs4251846	T	-	-	-	-	-	-	-
rs2239374	T	-	-	-	-	-	-	-
rs2286960	T	-	Slowed	-	-	-	-	-
rs4251805	A	-	-	-	-	-	-	-
rs344781	C	-	NA	-	-	-	-	-
rs2356338	T	Risk	Accelerated	Increased	-	-	-	-
rs344780	T	-	-	-	-	-	-	-
rs344779	T	-	-	-	-	-	-	Increased
rs8113334	C	--	-	-	-	-	-	-
rs4493171	T	Protection	-	Decreased	-	-	-	-
rs7259340	A	-	-	-	-	-	-	-
rs11668247	T	-	-	-	-	Protection	-	Increased
rs346043	C	Risk	Accelerated	Increased	-	-	-	-
rs740587	C	-	-	-	Protection	-	-	Increased
rs346054	G	-	-	-	-	-	-	-

*Significant Family Based Association Test (FBAT) results from our previous study.[3] using three asthma family cohorts from Nottingham, Southampton and The Netherlands (n = 587 families). The asthma patients that were probands of the Dutch asthma families (n = 200) were longitudinally followed in the Netherlands for approximately 30 years (1965-1995). FEV_1 _decline over time was modelled. FEV_1 _forced expiratory volume in one second; FVC forced vital capacity. NA = Not available.

In our first analyses we determined if *PLAUR *SNPs are risk factors for the development of spirometry defined COPD. These data identified only one SNP association (p < 0.01) *i.e*. rs740587 in the 5'region was protective however multiple borderline associations were observed particularly for additional SNPs in the promoter/5'region. We have previously shown that asthma risk alleles span the entire *PLAUR *gene including the 5'region, intron 3 and 3'region (Table 4, [3]). There was no direct concordance between asthma and COPD risk alleles and our data does not support the conclusion that *PLAUR *SNPs are major risk factors for the development of COPD in smokers.

The finding that the only signal for COPD susceptibility maps to the 5'region is of interest due to the observation that soluble PLAUR is elevated seven fold in the sputum of COPD patients [16] suggesting mechanisms underlying disease association may at least in part involve altered *PLAUR *transcription. In agreement with this hypothesis a recent study identified PLAUR mRNA expression in lung tissue as a marker of COPD and showed a significant correlation between FEF_25-75_(%Pred) and PLAUR mRNA expression (r = -0.44) [17].

COPD severity analyses based on GOLD classification did not identify any SNP showing association. Additional analysis of extremes of severity (GOLD 1 versus 4) again highlighted a role for rs11668247 in the 5'region in keeping with the COPD susceptibility analyses suggesting a modest influence of the distal 5'region SNPs.

In order to extend our case/control analyses we investigated the role of *PLAUR *SNPs in determining baseline lung function in this cohort. These analyses identified several significant associations; rs2302524(Lys220Arg) and rs2283628 (intron 3) were associated with lower and higher FEV_1 _respectively. rs740587(5'UTR-22346), rs11668247(5'UTR-20040) and rs344779(5'UTR-3666) in the 5'region were all associated with increased FEV_1_/FVC ratio. Interestingly, several SNPs showed very clear allele dose effects on FEV_1_/FVC ratio *e.g*. rs740587(5'UTR-22346), providing greater confidence in these data. The magnitude of effect was ~5% change in FEV_1_/FVC between genotypes and so potentially these effects are clinically relevant and surprising for a single SNP in a single gene. Interestingly, two of the SNPs associated with baseline FEV_1_/FVC were also associated with COPD susceptibility and severity respectively, *i.e*. rs740587(5'UTR-22346) and rs11668247(5'UTR-20040) in the 5'region. The rs2302524 (Lys220Arg) association with reduced baseline FEV_1 _values is of interest as this variant may be predicted to change the coding sequence of the PLAUR protein and this polymorphism has previously been associated with increased asthma risk and increased decline in FEV_1 _in asthma ([3], Table 4) The Lys220Arg is considered a conservative substitution, however the functional significance of this finding remains to be resolved. Interestingly, our data suggests a potential need for both alleles to be present prior to a physiological effect, *i.e*. a recessive effect.

It is also important to note that the single SNP associations reflect the linkage disequilibrium spanning the *PLAUR *gene *e.g*. rs11668247 and rs740587 are both associated with increased baseline FEV_1_/FVC and these SNPs are in high LD *i.e*. are inherited together more than by chance suggesting they both tag or contribute to the causation mechanism.

For the key associations with lung function we were also interested to determine if males or females were driving the associations and therefore completed gender specific analyses. All baseline features including mean age, smoking pack years and lung function (pre and post bronchodilator FEV_1 _and FEV_1_/FVC) were significantly different between the male and female smokers. These data are in keeping with a recent study that examined gender specific differences between subjects with severe emphysema and again identified less severe airway obstruction in females [18]. Our data suggest that the rs740587(5'UTR-22346) and rs344779(5'UTR-3666) associations with FEV_1_/FVC are driven by the male subjects as females do not exhibit the allele dose effect on FEV_1_/FVC ratio. Interesting, by selecting male subjects only the magnitude of effect was also increased *i.e*. the range of FEV_1_/FVC between genotypes for rs344779 for all subjects was 5.1% but for males was 7.3%, females 2.7%. This would suggest females do not demonstrate the lower FEV_1_/FVC ratio in carriers of the common alleles at these loci. These data are intriguing and potentially suggest that the effects of the polymorphisms in the 5'region are unmasked only in males *i.e*. gender specific transcriptional mechanisms. However, it is also feasible that the SNP effects are only identifiable in more severe airway obstruction *i.e*. in the males. This observation requires further investigation.

Cardiovascular disease including; ischemic heart diseases, hypertension and myocardial infarction have also been shown to be a common co-morbidity in COPD [11] and COPD may be an independent risk factor for the development of cardiovascular disease [19]. *PLAUR *levels have been associated with vascular remodelling [20] and monocyte adhesion in acute myocardial infarction [21]. Therefore it is feasible that in the COPD case control analyses we are really testing association with cardiovascular disease not COPD. While we have not formally tested the association between *PLAUR *SNPs and cardiovascular disease outcomes as we do not have this information, this seems unlikely. Our data suggests *PLAUR *SNPs influence baseline lung function in smokers irrespective of the presence or absence of COPD. Similarly, we have identified that *PLAUR *SNPs are determinants of baseline lung function in asthmatic children where clearly cardiovascular disease is not anticipated to confound the analyses [3].

In order to begin to investigate mechanisms underlying associations we have sequenced 4000 bp of the core promoter and identified a further five novel and two reported polymorphisms making a total of 12 polymorphisms in high LD (Additional file 3). Several of these SNPs generate ets family binding sites. c-ets-1 is of particular interest as this transcription factor is induced by oxidative stress *e.g*. H_2_O_2 _[22]. Importantly, H_2_O_2 _in breath condensate is elevated in COPD [23] and asthma [24]. These data potentially provide the link between the airway environment and expression of PLAUR determined by the presence/absence of SNPs. Interestingly, PLAU and PAI-1 expression are also elevated in sputum from asthma and COPD subjects [16] suggesting the plasminogen system may be augmented in these diseases. Importantly, *PLAU *and *PAI1 *transcription is also up regulated by ets-1 [25,26]. Also of interest is the alteration in Hypoxia Inducible Factor (HIF) binding sites due to the presence of a polymorphism (*e.g*. SNP rs11668247(5'UTR-20040) that was associated with COPD severity and lung function generates a HIF1 site. *PLAUR *transcription has been shown to be increased by HIF1β under hypoxia conditions [27] which may be a mechanism involved in epithelial mesenchymal transition (EMT) [28].

**Figure 3:**
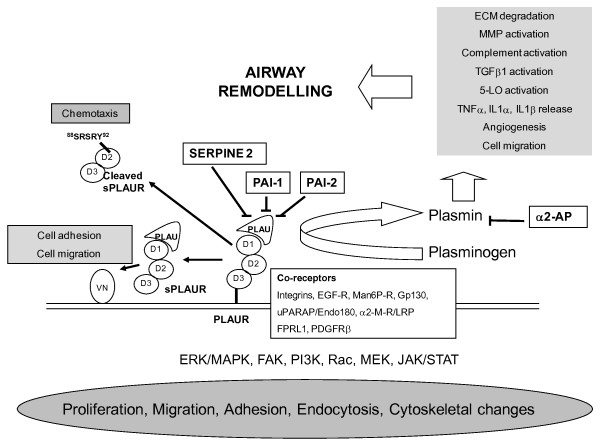
**The complex role of the PLAU-PLAUR pathway in the airways**. PLAUR is a complex, multi-domain (D1, 2, 3) molecule and exists as a membrane bound GPI linked protein and in multiple soluble forms. The interaction between PLAU-PLAUR is critical for the conversion of plasminogen to plasmin and is regulated by a series of proteins including; PAI-1, PAI-2 and SERPINE2 which have previously been implicated in COPD pathogenesis [16,31]. Plasmin has many downstream proteolytic effects including those common to remodelling of the airways *e.g*. MMP activation. In addition PLAUR interacts with several membrane receptors leading to the activation of signalling cascades resulting in alterations in; proliferation, migration, adhesion, endocytosis and cytoskeletal changes. PLAUR also exists in multiple soluble forms (sPLAUR) generated by splicing and/or proteolytic cleavage implicated in chemotaxis and interactions with the extracellular matrix [5,35,36].

The exact mechanism by which PLAUR contributes to the underlying pathobiology of the airways remains to be resolved, however its role in the generation of plasmin which subsequently leads to ECM degradation, MMP activation, TGFβ1 activation, 5-lipoxygenase activation and cell migration implicate tissue remodelling as a key function [29,30]. The complex role of the PLAU-PLAUR pathway in the airways is illustrated in Figure 3. It is important to note that there is accumulating evidence that genetic polymorphism in genes encoding components of the PLAU-PLAUR regulatory network constitute risk factors for the development of COPD *e.g*. polymorphisms spanning Serpin peptidase inhibitor, clade E (*SERPINE2*) have shown significant association with COPD susceptibility, FEV_1 _and FEV_1_/FVC [31]. The *SERPINE2 *gene encodes a PLAU inhibitor providing further support for the role of the PLAU-PLAUR pathway in COPD pathogenesis.

Chromosome 19q13 has previously been investigated for genetic determinants influencing COPD susceptibility. In particular one of five tested SNPs spanning Transforming Growth Factor β1 *TGFB1 *(at 41.8 Mbp) was associated with severe COPD in case (n = 304) control (n = 441) analyses and airway obstruction in family (77 pedigrees) analyses (p < 0.05) [10]. Similarly, one of four tested SNPs within latent transforming growth factor beta binding protein 4, *LTB4P *(at 41.8 Mbp) was associated (p = 0.01) with a densiometric emphysema distribution phenotype (Basal 1/3 lower lobe) [32] but not lung function [33] in 282 and 304 subjects respectively from the National Emphysema Trial. Overall, therefore the evidence that *TGFB1 *and *LTB4P *underlie the linkage with FEV_1 _and FEV_1_/FVC observed to the region is underwhelming. Importantly, in our asthma analyses we excluded these regions as containing the main dominants of lung function using a combination of linkage and association analyses [3]. However, it is also important to note that in COPD the linkage peak may involve genetic determinants in multiple genes underlying the signal.

This study extends our asthma association and has several strengths including extensive evaluation of polymorphic variation spanning the *PLAUR *region to examine COPD susceptibility and the examination of dichotomous and continual lung function traits. However, the limitations of this study include the absence of an independent replication sample, and that some of the dichotomous analyses were based on small numbers. The low number of controls versus cases in the dichotomous COPD analysis may at least in part explain the lack of concordance in findings generated using the continual lung function traits which provided more clear evidence for association. It is also important to note that the current study examines the role of *PLAUR *SNPs in the development of airway obstruction in smokers and does not test the role of *PLAUR *polymorphisms in non smoking individuals.

## Conclusion

We provide preliminary evidence that *PLAUR *SNPs influence baseline lung function in smokers. However, our data does not support a significant role for *PLAUR *SNPs contributing to the multiple genetic factors that predispose smokers to develop COPD.

## Competing interests

The authors declare that they have no competing interests.

## Authors' contributions

IS designed the study, completed the statistical analyses and drafted the manuscript. CES completed the gene sequencing and promoter analyses. IPH, SGH, MFM, AJW, MJC and CR recruited and clinically characterised subjects. All authors contributed to the final version of the manuscript.

## Pre-publication history

The pre-publication history for this paper can be accessed here:

http://www.biomedcentral.com/1471-2350/10/112/prepub

## Supplementary Material

Additional file 1 **COPD Severity Analyses (GOLD classification)**. This file contains phenotypic characteristics of GOLD stratified COPD subjects and association analyses for *PLAUR *SNPs with disease severity. Click here for file

Additional file 2 **Baseline Lung Function Analyses**. This file contains details of linear regression analyses for baseline lung function (FEV_1_, FEV_1_/FVC) for *PLAUR *SNPs. Click here for file

Additional file 3 ***PLAUR *5'region sequencing and bioinformatics analyses**. This file contains details of additional sequencing of the *PLAUR *promoter region including the identification of novel SNPs and a bioinformatics analysis to identify putative transcription factor changes resulting from SNPs. Click here for file
