# Supplementary material for: PLAUR polymorphisms and lung function in UK smokers
Source: BMC Med Genet. 2009 Oct 31;10:112. doi: 10.1186/1471-2350-10-112 (PMC2784766; doi:10.1186/1471-2350-10-112)
Supplement: Additional file 1 — COPD Severity Analyses (GOLD classification). This file contains phenotypic characteristics of GOLD stratified COPD subjects and association analyses for PLAUR SNPs with disease severity. [file 1471-2350-10-112-S1.doc]

**TABLE 1** Phenotypic characteristics of GOLD stratified COPD subjects (n=643)

|  | **GOLD 1** | **GOLD 2** | **GOLD 3** | **GOLD 4** | **Comparison** |
| --- | --- | --- | --- | --- | --- |
| Age (mean±SD) | 62.5±9.9 | 63.7±9.4 | 67.4±8.6 | 67.0±8.5 | p<0.0001 |
| Female (%) | 46.5 | 44.2 | 40.2 | 34.5 | p=ns |
| Smoking pack years (mean±SD) | 45.9±23.9 | 47.5±26.1 | 47.35±28.4 | 47.90±23.8 | p=ns |
| Baseline FEV_1_ % predicted (mean±SD) | 83.07±8.10 | 56.79±11.27 | 35.50±6.50 | 22.20±4.38 | p<0.0001 |
| Baseline FEV_1_/FVC ratio (mean±SD) | 62.2±7.3 | 55.1±9.6 | 43.9±10.2 | 36.6±11.6 | p<0.0001 |
| Change in FEV_1_ % predicted (salbutamol, mean±SD) | 5.86±5.99 | 5.31±7.10 | 4.59±4.47 | 2.08±3.20 | p<0.0001 |
| Number | 44 | 209 | 273 | 117 |  |

Subjects (where data available) were stratified using post bronchodilator lung function,

- GOLD 1 (post BD FEV_1_>80% pred, FEV_1_/FVC<0.7)
- GOLD 2 (post BD FEV_1_50-80% pred, FEV_1_/FVC<0.7)
- GOLD 3 (post BD FEV_1_30-50%, pred FEV_1_/FVC<0.7)
- GOLD 4 (post BD FEV_1_<30% pred, FEV_1_/FVC<0.7)

Continual variables between groups were compared by ANOVA, categorical variables by Pearson chi square.

|  | GOLD 1 (n=43) |  |  |  | GOLD 2 (n=206) |  |  |  | GOLD 3 (n=267) |  |  |  | GOLD 4 (n=115) |  |  |  | p-value | p-value |
| --- | --- | --- | --- | --- | --- | --- | --- | --- | --- | --- | --- | --- | --- | --- | --- | --- | --- | --- |
| SNP/Allele | 0 | 1 | 2 | MAF | 0 | 1 | 2 | MAF | 0 | 1 | 2 | MAF | 0 | 1 | 2 | MAF | (genotype) | (allele) |
| rs4803648 | 24 | 18 | 1 | 0.23 | 133 | 62 | 9 | 0.20 | 168 | 86 | 11 | 0.20 | 78 | 33 | 2 | 0.16 | 0.6111 | 0.4886 |
| rs4802189 | 26 | 16 | 1 | 0.21 | 146 | 54 | 5 | 0.16 | 188 | 72 | 7 | 0.16 | 84 | 26 | 1 | 0.13 | 0.6285 | 0.3272 |
| rs4251953 | 37 | 4 | 0 | 0.05 | 186 | 20 | 0 | 0.05 | 248 | 17 | 2 | 0.04 | 106 | 6 | 0 | 0.03 | 0.4657 | 0.5889 |
| rs4251938 | 31 | 11 | 1 | 0.15 | 166 | 36 | 4 | 0.11 | 206 | 59 | 5 | 0.13 | 92 | 24 | 0 | 0.10 | 0.6109 | 0.4989 |
| rs4251923 | 42 | 2 | 0 | 0.02 | 189 | 15 | 0 | 0.04 | 247 | 21 | 0 | 0.04 | 108 | 8 | 0 | 0.03 | 0.8879 | 0.8934 |
| rs4760 | 26 | 16 | 0 | 0.19 | 152 | 50 | 4 | 0.14 | 201 | 62 | 4 | 0.13 | 78 | 32 | 5 | 0.18 | 0.1699 | 0.1888 |
| rs2302524 | 29 | 14 | 1 | 0.18 | 153 | 49 | 5 | 0.14 | 181 | 75 | 10 | 0.18 | 86 | 26 | 2 | 0.13 | 0.6185 | 0.2616 |
| rs4251864 | 32 | 10 | 2 | 0.16 | 176 | 26 | 3 | 0.08 | 234 | 36 | 1 | 0.07 | 98 | 18 | 1 | 0.09 | 0.1193 | 0.0436 |
| rs2239372 | 11 | 20 | 9 | 0.48 | 45 | 105 | 57 | 0.53 | 72 | 125 | 71 | 0.50 | 33 | 53 | 29 | 0.48 | 0.8337 | 0.6154 |
| rs2283628 | 27 | 16 | 0 | 0.19 | 137 | 58 | 11 | 0.19 | 187 | 74 | 10 | 0.17 | 72 | 40 | 2 | 0.19 | 0.2785 | 0.8476 |
| rs4251846 | 34 | 9 | 0 | 0.10 | 166 | 40 | 3 | 0.11 | 203 | 61 | 5 | 0.13 | 85 | 31 | 0 | 0.13 | 0.5049 | 0.6687 |
| rs2239374 | 31 | 10 | 2 | 0.16 | 141 | 63 | 3 | 0.17 | 182 | 74 | 11 | 0.18 | 80 | 34 | 1 | 0.16 | 0.4084 | 0.8681 |
| rs2286960 | 20 | 19 | 3 | 0.30 | 129 | 73 | 5 | 0.20 | 151 | 101 | 18 | 0.25 | 67 | 41 | 8 | 0.25 | 0.2641 | 0.1287 |
| rs4251805 | 41 | 3 | 0 | 0.03 | 184 | 19 | 0 | 0.05 | 246 | 22 | 0 | 0.04 | 108 | 5 | 1 | 0.03 | 0.3108 | 0.7834 |
| rs344781 | 25 | 13 | 5 | 0.27 | 116 | 75 | 12 | 0.24 | 174 | 77 | 13 | 0.20 | 68 | 39 | 8 | 0.24 | 0.3378 | 0.1953 |
| rs2356338 | 25 | 15 | 3 | 0.24 | 93 | 104 | 10 | 0.30 | 134 | 115 | 20 | 0.29 | 57 | 50 | 8 | 0.29 | 0.4946 | 0.7843 |
| rs344780 | 24 | 11 | 5 | 0.26 | 111 | 72 | 12 | 0.25 | 166 | 71 | 12 | 0.19 | 64 | 37 | 7 | 0.24 | 0.2348 | 0.162 |
| rs344779 | 17 | 18 | 8 | 0.40 | 83 | 96 | 28 | 0.37 | 100 | 135 | 32 | 0.37 | 45 | 57 | 14 | 0.37 | 0.8771 | 0.9652 |
| rs8113334 | 26 | 14 | 3 | 0.23 | 133 | 67 | 8 | 0.20 | 186 | 74 | 8 | 0.17 | 78 | 31 | 8 | 0.20 | 0.4457 | 0.3708 |
| rs4493171 | 29 | 14 | 1 | 0.18 | 123 | 74 | 10 | 0.23 | 156 | 101 | 11 | 0.23 | 70 | 38 | 6 | 0.22 | 0.9307 | 0.7905 |
| rs7259340 | 11 | 23 | 8 | 0.46 | 99 | 88 | 21 | 0.31 | 105 | 123 | 34 | 0.36 | 46 | 52 | 15 | 0.36 | 0.2226 | 0.0474 |
| rs11668247 | 8 | 25 | 9 | 0.51 | 93 | 84 | 32 | 0.35 | 98 | 131 | 40 | 0.39 | 49 | 51 | 17 | 0.36 | 0.0738 | 0.0467 |
| rs346043 | 24 | 17 | 3 | 0.26 | 101 | 95 | 11 | 0.28 | 145 | 108 | 17 | 0.26 | 60 | 47 | 8 | 0.27 | 0.9031 | 0.9168 |
| rs740587 | 9 | 23 | 11 | 0.52 | 80 | 87 | 40 | 0.40 | 83 | 135 | 49 | 0.44 | 43 | 48 | 23 | 0.41 | 0.2111 | 0.2042 |
| rs346054 | 13 | 21 | 9 | 0.45 | 67 | 106 | 34 | 0.42 | 71 | 132 | 64 | 0.49 | 31 | 58 | 28 | 0.49 | 0.5063 | 0.1835 |

**TABLE 2** GOLD stage and *PLAUR* SNPs (unadjusted)

0, 1, 2 represent number of genotypes for major, heterozygote and minor genotypes respectively.

**TABLE 3**  GOLD stage 1 versus 4 and *PLAUR* SNPs

|  | GOLD 1 (n=43) |  |  | GOLD 4  (n=115) |  |  |  | **Additive** |  |  | **Dominant** |  |
| --- | --- | --- | --- | --- | --- | --- | --- | --- | --- | --- | --- | --- |
| SNP | 0 | 1 | 2 | 0 | 1 | 2 | p-value | Odds ratio | 95%CI | p-value | Odds ratio | 95%CI |
| rs4803648 | 24 | 18 | 1 | 78 | 33 | 2 | 0.176 | 0.63 | 0.32-1.2 | 0.131 | 0.57 | 0.27-1.19 |
| rs4802189 | 26 | 16 | 1 | 84 | 26 | 1 | 0.088 | 0.54 | 0.26-1.10 | 0.080 | 0.50 | 0.23-1.08 |
| rs4251953 | 37 | 4 | 0 | 106 | 6 | 0 | 0.593 | 0.69 | 0.18-2.69 | 0.593 | 0.69 | 0.18-2.69 |
| rs4251938 | 31 | 11 | 1 | 92 | 24 | 0 | 0.231 | 0.62 | 0.28-1.36 | 0.309 | 0.65 | 0.29-1.49 |
| rs4251923 | 42 | 2 | 0 | 108 | 8 | 0 | 0.714 | 1.35 | 0.27-6.76 | 0.718 | 1.35 | 0.27-6.76 |
| rs4760 | 26 | 16 | 0 | 78 | 32 | 5 | 0.362 | 0.73 | 0.37-1.44 | 0.138 | 0.55 | 0.25-1.21 |
| rs2302524 | 29 | 14 | 1 | 86 | 26 | 2 | 0.258 | 0.67 | 0.34-1.34 | 0.211 | 0.61 | 0.28-1.32 |
| rs4251864 | 32 | 10 | 2 | 98 | 18 | 1 | 0.100 | 0.54 | 0.26-1.13 | 0.175 | 0.56 | 0.24-1.23 |
| rs2239372 | 11 | 20 | 9 | 33 | 53 | 29 | 0.801 | 1.07 | 0.64-1.78 | 0.972 | 1.02 | 0.45-2.31 |
| rs2283628 | 27 | 16 | 0 | 72 | 40 | 2 | 0.676 | 1.17 | 0.57-2.40 | 0.781 | 1.11 | 0.53-2.36 |
| rs4251846 | 34 | 9 | 0 | 85 | 31 | 0 | 0.571 | 1.28 | 0.54-3.02 | 0.571 | 1.28 | 0.54-3.02 |
| rs2239374 | 31 | 10 | 2 | 80 | 34 | 1 | 0.818 | 0.92 | 0.46-1.85 | 0.864 | 1.07 | 0.49-2.36 |
| rs2286960 | 20 | 19 | 3 | 67 | 41 | 8 | 0.360 | 0.71 | 0.34-1.50 | 0.362 | 0.71 | 0.35-1.47 |
| rs4251805 | 41 | 3 | 0 | 108 | 5 | 1 | 0.861 | 1.12 | 0.31-4.14 | 0.952 | 0.96 | 0.22-4.20 |
| rs344781 | 25 | 13 | 5 | 68 | 39 | 8 | 0.513 | 0.83 | 0.48-1.44 | 0.815 | 0.92 | 0.45-1.89 |
| rs2356338 | 25 | 15 | 3 | 57 | 50 | 8 | 0.573 | 1.19 | 0.66-2.15 | 0.404 | 1.36 | 0.66-2.80 |
| rs344780 | 24 | 11 | 5 | 64 | 37 | 7 | 0.550 | 0.84 | 0.47-1.50 | 0.996 | 1.00 | 0.47-2.15 |
| rs344779 | 17 | 18 | 8 | 45 | 57 | 14 | 0.474 | 0.825 | 0.49-1.40 | 0.936 | 0.97 | 0.47-2.02 |
| rs8113334 | 26 | 14 | 3 | 78 | 31 | 8 | 0.494 | 0.82 | 0.46-1.45 | 0.455 | 0.75 | 0.36-1.58 |
| rs4493171 | 29 | 14 | 1 | 70 | 38 | 6 | 0.439 | 1.29 | 0.68-2.45 | 0.525 | 1.27 | 0.61-2.68 |
| rs7259340 | 11 | 23 | 8 | 46 | 52 | 15 | 0.137 | 0.67 | 0.39-1.14 | 0.144 | 0.55 | 0.25-1.22 |
| **rs11668247** | 8 | 25 | 9 | 49 | 51 | 17 | **0.023** | **0.54** | **0.32-0.92** | **0.011** | **0.32** | **0.14-0.77** |
| rs346043 | 24 | 17 | 3 | 60 | 47 | 8 | 0.836 | 1.06 | 0.60-1.90 | 0.709 | 1.15 | 0.56-2.33 |
| rs740587 | 9 | 23 | 11 | 43 | 48 | 23 | 0.115 | 0.67 | 0.41-1.10 | 0.052 | 0.44 | 0.19-1.01 |
| rs346054 | 13 | 21 | 9 | 31 | 58 | 28 | 0.587 | 1.15 | 0.70-1.89 | 0.743 | 1.14 | 0.52-2.50 |

0, 1, 2 represent number of genotypes for major, heterozygote and minor genotypes respectively. Logistic regression was used to compare genotype frequencies using the additive and dominant models with covariate age included in the model. The recessive model was not investigated due to low numbers of subjects.
