# Supplementary material for: PLAUR polymorphisms and lung function in UK smokers
Source: BMC Med Genet. 2009 Oct 31;10:112. doi: 10.1186/1471-2350-10-112 (PMC2784766; doi:10.1186/1471-2350-10-112)
Supplement: Additional file 2 — Baseline Lung Function Analyses. This file contains details of linear regression analyses for baseline lung function (FEV1, FEV1/FVC) for PLAUR SNPs. [file 1471-2350-10-112-S2.doc]

**TABLE 1 Baseline lung function (FEV_1_) and *PLAUR*** SNPs

|  | Smokers | (n=992) |  |  | **Additive** |  |  | **Recessive** |  |  | **Dominant** |  |
| --- | --- | --- | --- | --- | --- | --- | --- | --- | --- | --- | --- | --- |
| SNP | 0 | 1 | 2 | p-value | group | Value (l, SE) | p-value | group | Value (l, SE) | p-value | group | Value (l, SE) |
| rs4803648 | 577 | 292 | 36 | 0.980 | - | - | 0.851 | - | - | 0.904 | - | - |
| rs4802189 | 629 | 249 | 22 | 0.959 | - | - | 0.849 | - | - | 0.798 | - | - |
| rs4251953 | 840 | 72 | 3 | 0.354 | - | - | 0.848 | - | - | 0.172 | - | - |
| rs4251938 | 709 | 201 | 13 | 0.495 | - | - | 0.347 | - | - | 0.362 | - | - |
| rs4251923 | 859 | 63 | 0 | 0.381 | - | - | ND | - | - | 0.381 | - | - |
| rs4760 | 644 | 234 | 27 | 0.596 | - | - | 0.376 | - | - | 0.470 | - | - |
| rs2302524 | 647 | 254 | 22 | **0.007** | 0  1  2 | 1.54±0.03  1.62±0.04  1.14±0.15 | **0.006** | 0/1  2 | 1.56±0.02  1.14±0.15 | 0.394 | - | - |
| rs4251864 | 774 | 144 | 9 | 0.735 | - | - | 0.466 | - | - | 0.665 | - | - |
| rs2239372 | 233 | 450 | 234 | 0.853 | - | - | 0.597 | - | - | 0.994 | - | - |
| rs2283628 | 626 | 258 | 40 | 0.015 | 0  1  2 | 1.55±0.03  1.50±0.04  1.84±0.11 | **0.008** | 0/1  2 | 1.54±0.03  1.84±0.11 | 0.816 | - | - |
| rs4251846 | 706 | 307 | 13 | 0.117 | - | - | 0.241 | - | - | 0.156 | - | - |
| rs2239374 | 614 | 282 | 26 | 0.488 | - | - | 0.595 | - | - | 0.242 | - | - |
| rs4251831 | 453 | 406 | 54 | 0.249 | - | - | 0.414 | - | - | 0.107 | - | - |
| rs2286960 | 523 | 351 | 49 | 0.288 | - | - | 0.352 | - | - | 0.330 | - | - |
| rs4251805 | 851 | 66 | 1 | 0.255 | - | - | ND | - | - | 0.458 | - | - |
| rs344781 | 543 | 315 | 54 | 0.744 | - | - | 0.442 | - | - | 0.833 | - | - |
| rs2356338 | 465 | 397 | 61 | 0.445 | - | - | 0.916 | - | - | 0.211 | - | - |
| rs344780 | 520 | 286 | 50 | 0.546 | - | - | 0.273 | - | - | 0.805 | - | - |
| rs344779 | 332 | 457 | 133 | 0.247 | - | - | 0.235 | - | - | 0.137 | - | - |
| rs8113334 | 602 | 287 | 37 | 0.754 | - | - | 0.526 | - | - | 0.573 | - | - |
| rs4493171 | 552 | 331 | 38 | 0.404 | - | - | 0.186 | - | - | 0.925 | - | - |
| rs7259340 | 372 | 423 | 114 | 0.543 | - | - | 0.938 | - | - | 0.284 | - | - |
| rs1994417 | 441 | 373 | 109 | 0.862 | - | - | 0.587 | - | - | 0.879 | - | - |
| rs11668247 | 350 | 422 | 155 | 0.163 | - | - | 0.092 | - | - | 0.156 | - | - |
| rs346043 | 499 | 367 | 53 | 0.599 | - | - | 0.770 | - | - | 0.311 | - | - |
| rs740587 | 292 | 430 | 198 | 0.107 | - | - | 0.102 | - | - | 0.065 | - | - |
| rs346054 | 283 | 446 | 196 | 0.401 | - | - | 0.737 | - | - | 0.265 | - | - |

Regression analysis was used to investigate the association between *PLAUR* SNPs and baseline FEV_1_ using the additive, recessive or dominant models. Covariates included in the model were age, gender, height and pack years. ND = not determined due to low numbers. Associations with a p<0.01 are shown in bold.

**TABLE 2 Baseline lung function (FEV_1_/FVC) and *PLAUR* SNPs**

|  | Smokers | (n=992) |  |  | **Additive** |  |  | **Recessive** |  |  | **Dominant** |  |
| --- | --- | --- | --- | --- | --- | --- | --- | --- | --- | --- | --- | --- |
| SNP | 0 | 1 | 2 | p-value | group | Value (%, SE) | p-value | group | Value (%, SE) | p-value | group | Value (%, SE) |
| rs4803648 | 575 | 289 | 36 | 0.347 | - | - | 0.336 | - | - | 0.189 | - | - |
| rs4802189 | 629 | 249 | 22 | 0.237 | - | - | 0.604 | - | - | 0.091 | - | - |
| rs4251953 | 835 | 72 | 3 | 0.494 | - | - | 0.883 | - | - | 0.236 | - | - |
| rs4251938 | 707 | 198 | 13 | 0.451 | - | - | 0.960 | - | - | 0.214 | - | - |
| rs4251923 | 854 | 63 | 0 | 0.831 | - | - | ND | - | - | 0.831 | - | - |
| rs4760 | 640 | 233 | 27 | 0.365 | - | - | 0.227 | - | - | 0.293 | - | - |
| rs2302524 | 647 | 254 | 22 | 0.041 | 0  1  2 | 55.0±0.6  57.8±1.0  52.3±3.4 | 0.308 | - | - | 0.046 | 0  1/2 | 55.0±0.6  57.4±1.0 |
| rs4251864 | 770 | 143 | 9 | 0.704 | - | - | 0.616 | - | - | 0.588 | - | - |
| rs2239372 | 232 | 446 | 134 | 0.997 | - | - | 0.963 | - | - | 0.942 | - | - |
| rs2283628 | 623 | 256 | 40 | 0.039 | 0  1  2 | 55.8±0.6  54.4±1.0  61.3±2.6 | 0.025 | 0/1  2 | 55.4±0.5  61.3±2.6 | 0.654 | - | - |
| rs4251846 | 704 | 204 | 13 | 0.303 | - | - | 0.342 | - | - | 0.346 | - | - |
| rs2239374 | 612 | 280 | 25 | 0.846 | - | - | 0.735 | - | - | 0.708 | - | - |
| rs4251831 | 450 | 404 | 54 | 0.022 | 0  1  2 | 57.0±0.8  54.8±0.8  51.8±0.2 | 0.067 | - | - | 0.014 | 0  1/2 | 57.0±0.8  54.4±0.8 |
| rs2286960 | 521 | 348 | 49 | 0.863 | - | - | 0.587 | - | - | 0.863 | - | - |
| rs4251805 | 846 | 66 | 1 | 0.168 | - | - | ND | - | - | 0.478 | - | - |
| rs344781 | 541 | 312 | 54 | 0.073 |  |  | 0.043 | 0/1  2 | 55.5±0.6  60.1±2.2 | 0.100 | - | - |
| rs2356338 | 462 | 395 | 61 | 0.114 | - | - | 0.433 | - | - | 0.043 | 0  1/2 | 56.7±0.8  54.5±0.8 |
| rs344780 | 519 | 283 | 50 | 0.228 |  |  | 0.131 | - | - | 0.209 | - | - |

**TABLE 2 Baseline lung function (FEV_1_/FVC) and *PLAUR* SNPs *continued***

|  | Smokers | (n=992) |  |  | **Additive** |  |  | **Recessive** |  |  | **Dominant** |  |
| --- | --- | --- | --- | --- | --- | --- | --- | --- | --- | --- | --- | --- |
| SNP | 0 | 1 | 2 | p-value | group | Value (%, SE) | p-value | group | Value (%, SE) | p-value | group | Value (%, SE) |
| rs344779 | 331 | 455 | 132 | **0.009** | 0  1  2 | 54.0±0.9  55.9±0.8  59.1±1.4 | **0.009** | 0/1  2 | 55.1±0.6  59.1±1.4 | 0.018 | 0  1/2 | 54.0±0.9  56.7±0.7 |
| rs8113334 | 600 | 284 | 37 | 0.201 | - | - | 0.356 | - | - | 0.083 | - | - |
| rs4493171 | 549 | 329 | 38 | 0.273 | - | - | 0.145 | - | - | 0.768 | - | - |
| rs7259340 | 371 | 419 | 114 | 0.387 | - | - | 0.168 | - | - | 0.686 | - | - |
| rs1994417 | 439 | 372 | 107 | 0.665 | - | - | 0.437 | - | - | 0.482 | - | - |
| rs11668247 | 349 | 418 | 155 | 0.014 | 0  1  2 | 54.5±0.9  55.4±0.8  59.0±1.3 | **0.005** | 0/1  2 | 55.0±0.6  59.0±1.3 | 0.095 | - | - |
| rs346043 | 494 | 367 | 53 | 0.115 | - | - | 0.514 | - | - | 0.038 | 0  1/2 | 56.7±0.7  54.4±0.8 |
| rs740587 | 292 | 426 | 197 | **0.003** | 0  1  2 | 53.5±0.9  55.8±0.8  58.5±1.1 | **0.005** | 0/1  2 | 54.9±0.6  58.5±1.1 | **0.005** | 0  1/2 | 53.5±0.9  56.7±0.6 |
| rs346054 | 281 | 443 | 196 | 0.366 | - | - | 0.658 | - | - | 0.266 | - | - |

Regression analysis was used to investigate the association between *PLAUR* SNPs and baseline FEV_1_/FVC using the additive, recessive or dominant models. Covariates included in the model were age, gender, height and pack years. Associations with a p<0.01 are shown in bold.

**TABLE 3 Gender specific analyses of baseline lung function (FEV_1_ and FEV_1_/FVC) and associated *PLAUR* SNPs**

|  | Phenotype |  | **ALL** |  |  | **MALES** |  |  | **FEMALES** |  |
| --- | --- | --- | --- | --- | --- | --- | --- | --- | --- | --- |
| SNP |  | p-value | Group (n) | Value, SE | p-value | Group (n) | Value, SE | p-value | Group (n) | Value, SE |
| rs2302524 | FEV_1_ | 0.007 | 0 (647)  1 (254)  2 (22) | 1.54±0.03  1.62±0.04  1.14±0.15 | 0.198 | 0 (360)  1 (143)  2 (11) | 1.69±0.04  1.69±0.07  1.26±0.24 | 0.002 | 0 (287)  1 (111)  2 (11) | 1.35±0.04  1.53±0.06  1.01±0.18 |
| rs2283628 | FEV_1_ | 0.015 | 0 (626)  1 (258)  2 (40) | 1.55±0.03  1.50±0.04  1.84±0.11 | 0.029 | 0 (350)  1 (144)  2 (24) | 1.70±0.42  1.57±0.07  2.00±0.16 | 0.255 | 0 (276)  1 (114)  2 (18) | 1.37±0.04  1.40±0.06  1.62±0.15 |
| rs344779 | FEV_1_/FVC | 0.009 | 0 (331)  1 (455)  2 (132) | 54.0±0.9  55.9±0.8  59.1±1.4 | 0.007 | 0 (185)  1 (260)  2 (66) | 51.8±1.2  54.5±1.0  59.1±2.0 | 0.531 | 0 (146)  1 (195)  2 (66) | 56.8±1.4  57.7±1.2  59.5±1.8 |
| rs11668247 | FEV_1_/FVC | 0.014 | 0 (349)  1 (418)  2 (155) | 54.5±0.9  55.4±0.8  59.0±1.3 | 0.059 | 0 (209)  1 (226)  2 (79) | 52.2±1.1  54.7±1.1  57.1±1.8 | 0.063 | 0 (140)  1 (192)  2 (76) | 57.7±1.4  56.2±1.2  61.4±1.8 |
| rs740587 | FEV_1_/FVC | 0.003 | 0 (292)  1 (426)  2 (197) | 53.5±0.9  55.8±0.8  58.5±1.1 | 0.001 | 0 (170)  1 (244)  2 (95) | 50.8±1.2  55.1±1.0  58.0±1.7 | 0.386 | 0 (122)  1 (182)  2 (102) | 57.1±1.4  56.7±1.2  59.4±1.6 |

Regression analysis was used to investigate the association between *PLAUR* SNPs and baseline FEV_1_ and FEV_1_/FVC in males and females using the additive model for SNPs previously associated (p<0.01 in any model) with lung function in Tables 1 and 2. Covariates included in the model were age, height and pack years.
