# Supplementary material for: PLAUR polymorphisms and lung function in UK smokers
Source: BMC Med Genet. 2009 Oct 31;10:112. doi: 10.1186/1471-2350-10-112 (PMC2784766; doi:10.1186/1471-2350-10-112)
Supplement: Additional file 3 — PLAUR 5'region sequencing and bioinformatics analyses. This file contains details of additional sequencing of the PLAUR promoter region including the identification of novel SNPs and a bioinformatics analysis to identify putative transcription factor changes resulting from SNPs. [file 1471-2350-10-112-S3.doc]

**TABLE 1** Summary of *PLAUR* transcription factor binding site changes found in two or more online database searches

| **SNP** | **19q Location**  **(Kbp)** | **Location in gene**  **(TSS)** | **Alleles** | **MAF** | **TF LOSS** | **TF GAIN** |
| --- | --- | --- | --- | --- | --- | --- |
|  |  |  |  |  |  |  |
| *novel 1 | 48866.144 | 5’UTR +18 | G/A | 0.03* (2/70) | no | no |
| rs4251805 | 48866.281 | 5’UTR -119 | G/A | 0.02 | no | no |
| rs344781 | 48866.628 | 5’UTR-466 | T/C | 0.20 | **E2F-1** | **Elk-1, c-ets-1, c-ets-2** |
| rs2356338 | 48866.811 | 5’UTR -649 | G/T | 0.26 | no | no |
| *novel 2 | 48867.161 | 5’UTR-999 | C/G | 0.03*(2/62) | no | no |
| *novel 3 | 48868.056 | 5’UTR-1894 | T/G | 0.26*(18/70) | no | **Elk-1, c-ets-1, c-ets-2** |
| *novel 4 | 48868.717 | 5’UTR-2555 | ^31bp Insertion | 0.30 (22/74) | no | **Elk-1, c-ets-1, c-ets-2,**  **Ik-2, XBP1, HOXA5** |
| rs11669095 | 48869.506 | 5’UTR-3344 | A/T | 0.26 (19/72) | **TBP, cdxA** | no |
| *novel 5 | 48869.540 | 5’UTR-3378 | C/T | 0.13 (9/70) | no | no |
| rs344780 | 48869.707 | 5’UTR-3544 | C/T | 0.18 | **IRF-1, IRF-2** | no |
| rs344779 | 48869.828 | 5’UTR-3665 | G/T | 0.40 | **YY1, CTF/NF-1, C/EBP, NF-Y** | no |
| rs344778 | 48869.977 | 5’UTR-3814 | G/A | 0.26 (19/72) | no | no |
| rs344772 | 48871.796 | 5’UTR-5633 | T/C | 0.37 | no | **C/EBP** |
| rs8113334 | 48873.035 | 5’UTR-6872 | T/C | 0.18 | no | no |
| rs4493171 | 48876.696 | 5’UTR-10533 | C/T | 0.20 | no | no |
| rs7259340 | 48878.493 | 5’UTR-12330 | C/A | 0.33 | no | no |
| rs11083715+ | 48882.211 | 5’UTR-16048 | C/T | 0.45 | no | no |
| rs11083716+ | 48882.502 | 5’UTR-16339 | G/A | 0.44 | no | no |
| rs11083718+ | 48884.315 | 5’UTR-18152 | G/A | 0.44 | no | **HIF-1, c-Myc** |
| rs346044+ | 48885.484 | 5’UTR-19321 | C/T | 0.21 | no | **AP1** |
| rs11668247 | 48886.202 | 5’UTR-20039 | C/T | 0.42 | **HIF1, c-Myc** | no |
| rs346043 | 48886.621 | 5’UTR-20458 | T/C | 0.24 | **C/EBP** | **c-Myb** |
| rs740587 | 48888.508 | 5’UTR-22345 | T/C | 0.46 | no | no |
| rs8110160+ | 48889.680 | 5’UTR-23517 | G/A | 0.24 | no | no |
| rs346054 | 48896.309 | 5’UTR-30146 | C/G | 0.43 | no | no |

Minor allele frequency (MAF) taken from NCBI (B36) except for *Novel SNPs identified by sequencing the first 4kb of the *PLAUR* promoter (including rs344772) in 37 Caucasian subjects (brackets show allele counts)

^31bp Insertion+ AAGGGATTCTCATGCAT[CAGCTTCCTGAGTAGTTGGGATTACAGGCAT]GCCTGGCTATTTTTTTTT

SNPs listed also include SNPs(+) in LD (HapMap v36) with those genotyped in the current analyses.
